# Impact of Neonatal Manipulation of Androgen Receptor Function on Endocrine-Metabolic Programming in the Juvenile Female Rat

**DOI:** 10.1155/2013/181950

**Published:** 2013-08-28

**Authors:** Luisina Ongaro, Andres Giovambattista, Eduardo Spinedi

**Affiliations:** ^1^Neuroendocrine Unit, IMBICE (CICPBA-CONICET La Plata), 1900 La Plata, Argentina; ^2^CENEXA (UNLP-CONICET La Plata, PAHO/WHO Collaborating Centre for Diabetes), Medical School, La Plata National University, Calles 60 y 120, 1900 La Plata, Argentina

## Abstract

The impact of neonatal androgen receptor (AR) stimulation/blockage, due to testosterone propionate (TP)/AR antagonist treatment, on individual anthropometry and neuroendocrine-metabolic function was evaluated in the juvenile female rat. Pups (age 5 days) were s.c. injected with TP (1.25 mg), flutamide (F; 1.75 mg), and TP + F or vehicle (control, CT) and studied on day 30 of age. Body weight (BW), parametrial adipose tissue (PMAT) mass, food intake, adipoinsular axis, and steroidogenic functions were examined. Opposite to TP-rats, F-treated rats developed hypophagia, grew slowly (BW and PMAT), and displayed heightened peripheral insulin sensitivity. These F effects were abrogated in TP + F animals. Accordingly, TP rats displayed hyperleptinemia, an effect fully prevented by F cotreatment. Finally, androgen-treated animals bore an irreversible ovarian dysfunction (reduced circulating levels of 17HOP4 and ovary 17HOP4 content and P450c17 mRNA abundance). These data indicate that early stimulation of AR enhanced energy store, blockage of AR activity resulted in some beneficial metabolic effects, and neonatally androgenized rats developed a severe ovarian dysfunction. Our study highlights the important role of AR in the early organizational programming of metabolic and neuroendocrine functions.

## 1. Introduction

Intrauterine and early postnatal endogenous and exogenous environments all have an important role for normal metabolic-neuroendocrine functions and neuronal development of the offspring [1]. Fetal sexual differentiation results from complex interplay between genetic and hormonal factors [2, 3], and testosterone playing a key role in these associated events is supported by the fact that excess testosterone in female fetuses results in functional, behavioral, and morphological defects in adulthood [2, 4, 5]. Insulin resistance is frequently associated with hyperandrogenemia, and clinical studies suggest that interaction between insulin and sex hormones takes place in healthy subjects [6]. Moreover, Barraclough CA [7] reported that just a single neonatal s.c. treatment with testosterone propionate (TP) in the female rat produces several metabolic and clinical features of the human polycystic ovary syndrome (PCOS). Moreover, we previously reported that transient androgenization in normal female rats at neonatal [8] or early postpubertal [9] ages did result in a seriously disrupted metabolic phenotype [10] accompanied by impaired peripheral and adipose tissue (AT) insulin sensitivity in adulthood.

Since biological active androgens exert their effects through binding to androgen receptor (AR) [11], the present study explored whether neonatal manipulation of AR function in normal female rat could alter metabolic and neuroendocrine functions when individuals reached juvenile age. For this objective, rats were neonatally treated with either testosterone [7], the nonsteroidal anti-AR flutamide [12], or both compounds in combination and then studied at 30 days of age. 

## 2. Materials and Methods

### 2.1. Experimental Animals

On day 5 of age, female rats (Sprague-Dawley; *n* = 12/16 per group obtained from 3-4 different litters) were s.c. injected with either 50 *μ*L of sterile corn oil alone (control; CT) or 1.25 mg of testosterone propionate (TP) [8], 1.75 mg flutamide (F) [13], or both (TP + F). Rats were checked daily (between days 7 and 30 of age) for vaginal opening. After weaning (age 21 days), rats were individually housed, and body weight (BW) and food intake were recorded daily (07:30–08:30 h) up to the experimental day (30 days of age). Animals were kept in a light (lights on 07:00 to 19:00 h) and temperature (22°C) controlled room. On the experimental day, animals were euthanized in nonfasting condition, and trunk blood was collected. Parametrial adipose tissue (PMAT) pads, adrenal glands (AGs), and ovaries were quickly dissected, weighed, and either homogenized or kept frozen (−80°C). Experiments followed international regulations concerning the ethical use of animals and were also approved by our Animal Care Committee. 

### 2.2. Metabolite Measurements

Plasma glucose (Wiener Argentina Lab.) and triglyceride (TG; Wiener) levels were determined by commercial kits. Circulating leptin [14], insulin [9], testosterone [15], 17*β*-estradiol (E2) [15], 5*α*-androstane-3*α*, 17*β*-diol (5*α*Diol) [16], and corticosterone [14] concentrations were determined by specific radioimmunoassays (RIAs) developed in our laboratories. (FULL TERM FIRST) HOMA values were calculated as [glucose (mg/dL) × insulin (ng/mL)]/405. Plasma levels of 17*α*-hydroxy-progesterone (17HOP4) were assayed by a commercial RIA kit (IMMUNOTECH SAS, France; standard curve ranging between 0.05–50 ng/mL; coefficients of variation intraassay and interassay were 7-8 and 5–15%, resp.). Tissues (AGs and ovaries) were either homogenized in a small volume (200–300 *μ*L) of 0.01 M HCl or kept frozen (−80°C) until assayed. The 10,000 ×g supernatants of tissue homogenates were then assayed for measurement of corticosterone (AGs) and 17HOP4 (AGs and ovaries), and values were expressed as mass of steroid per mg of protein (Wiener Lab.). 

### 2.3. Ovary RNA Isolation and Real-Time Quantitative PCR

Total RNA was isolated from ovaries of different groups by the single-step, acid guanidinium isothiocyanate-phenol-chloroform extraction method (Trizol; Invitrogen, Life Tech., USA; cat. # 15596-026) [17]. One *μ*g of total RNA was reverse-transcripted using random primers (250 ng) and Superscript III Rnase H-Reverse Transcriptase (200 U/*μ*L Invitrogen, Life Tech, USA; cat. # 18989-093). Primers applied: *β*-actin (ACTB) (R): 5′-ACCCTCATAGATGGGCACAG-3′, (F): 5′-AGCCATGTACGTAGCCATCC-3′ (115 pb) [GenBank Accession Number (GBAN): NM_031144] and cytochrome P450, family 17, subfamily A, polypeptide 1 (P450c17) (R): 5′-GGCCACGCCTTTCCCTTGGT-3′, (F): 5′-AGCTGGCCAGGGAGGTGCTC-3′ (101 pb) (GBAN: NM_012753.1). Two *μ*L of the reverse transcription mix was amplified with QuantiTect Syber Green PCR kit (Qiagen, cat. # 204143) containing 0.5 *μ*M of each specific primer, using LightCycler Detection System (MJ Mini Opticon, Biorad). PCR efficiency was ~1. Threshold cycles (Ct) were measured in separate tubes by duplicate. Identity and purity of the amplified product were checked by electrophoresis on agarose minigels, and the melting curve was analyzed at the end of amplification. The differences between the cycle threshold (Ct) were calculated in every sample for each gene of interest as follows: Ct gene of interest-Ct reporter gene. *β*-actin, whose mRNA levels did not differ comparing control to test groups, was the reporter gene. Relative changes in expression level of one specific gene (ΔΔCt) were calculated as ΔCt of the test group minus ΔCt of the control group and then presented as 2 − ΔΔCt.

### 2.4. Statistical Analysis

Data (means ± SEM) were analyzed by multiple-way ANOVA, followed by post hoc comparisons with Fisher's test or the nonparametric Mann-Whitney test [18]. *P* values under 0.05 were considered statistically different.

## 3. Results

### 3.1. Anthropometric Parameters

#### 3.1.1. Body Weight and Adipose Tissue Mass

**Figure 1:**
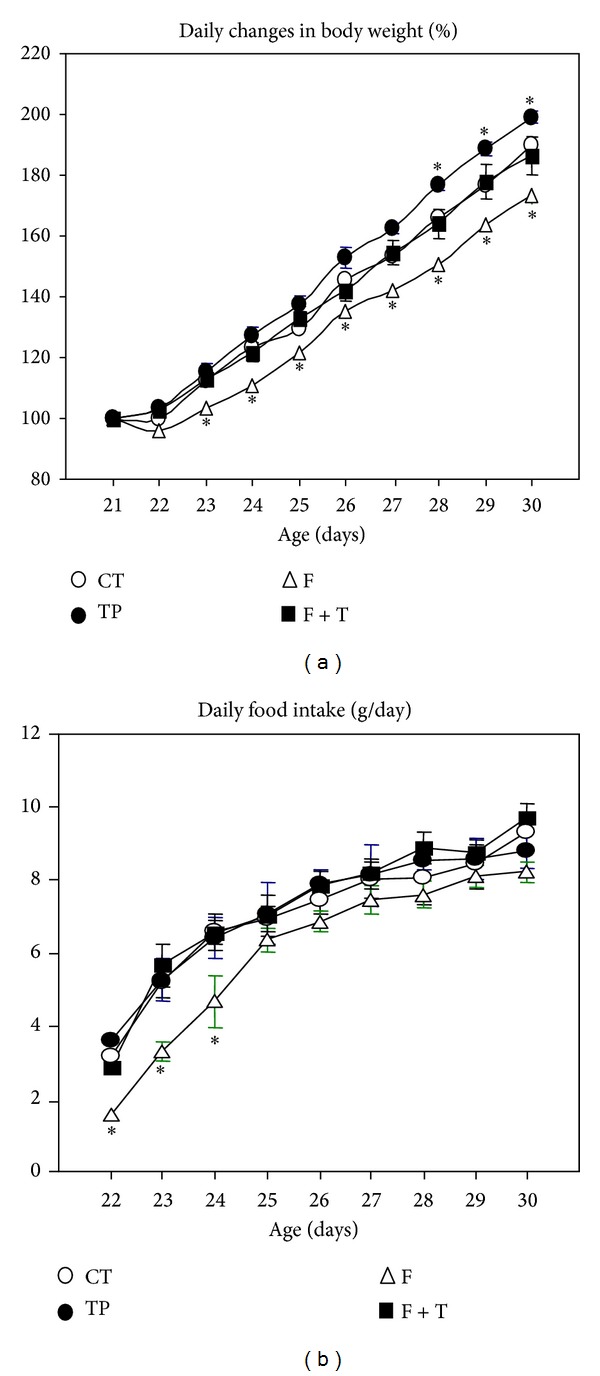
Changes in body weight (BW) (a) and food intake (b) in different groups of neonatally-treated rats, examined daily between weaning (age 21 days) and experimentation (age 30 days). Values are means ± SEM, *n* = 12/16 rats per group. *P < 0.05 versus CT values.

All experimental animals had comparable body weights at weaning [age 21 days; 30.46 ± 0.79, 31.56 ± 1.06, 29.65 ± 1.27 and 32.66 ± 1.57 g in CT (*n* = 12), TP (*n* = 16), F (*n* = 12) and TP + F (*n* = 16) groups, resp.]. On experimentation (age 30 days), F rats were the lightest (52.63 ± 1.95 g; *P* < 0.05 versus 58.75 ± 1.49 g in CT) and TP animals were the heaviest (63.38 ± 1.22 g; *P* < 0.05 versus CT), whereas BW values for TP + F rats (60.59 ± 0.72 g) only significantly (*P* < 0.05) differed from those of F group. No significant group differences in the slopes of growth curves were observed (data not shown); however, the curve displayed by F-treated rats was somewhat displaced to the right, and, conversely, that of TP-treated rats was somewhat displaced to the left (Figure 1(a)). Concordantly, the 9-day body weight catch-up (calculated between 21 and 30 days of age) was significantly (*P* < 0.05) lower in F (22.97 ± 1.11 g) than in CT (28.11 ± 0.78 g) rats, whereas those in TP (30.04 ± 1.01 g) and TP + F (27.93 ± 1.03 g) rats did not differ significantly from CT values. On the experimental day, only androgen-treated rats (TP and TP + F) displayed an open vagina; however, androgenized rats cotreated with F displayed a significant delay in the vaginal opening day (on 17 ± 0.9 days of age) compared to rats treated with TP alone (11.5 ± 1.5 days of age; *P* < 0.05). Individual PMAT pad mass was enlarged in TP rats (167.4 ± 8.3 versus 147.8 ± 9.7 mg/100 g BW in CT rats; *P* < 0.05) and reduced in F-treated animals (112.3 ± 8.5 mg/100 g BW; *P* < 0.05 versus CT); interestingly, this F effect was no longer observed when animals were cotreated with androgen (TP + F rats: 141.46 ± 13.5 mg/100 g BW). 

#### 3.1.2. Food Intake

Individual daily food intake (Figure 1(b)) was significantly (*P* < 0.05) lower in F rats (9 day-average: 6.01 ± 0.22 g/day) than in the remaining groups (7.01 ± 0.39, 7.14 ± 0.44, and 7.27 ± 0.29 g/day in CT, TP, and TP + F rats, resp.). Because of differences found in individual BW values of F-treated rats, the 9 day-average daily food intake was normalized by day after day change in rat BW and expressed in g/day/100 g BW. Data indicated that, although no differences were found between CT and TP/TP + F values, this difference in daily food intake remained the same in flutamide-treated rats: 16.34 ± 0.56 and 14.92 ± 0.35 g/day/100 g BW in CT and F animals, respectively (*P* < 0.05). 

### 3.2. Adipoinsular Axis Activity

**Figure 2:**
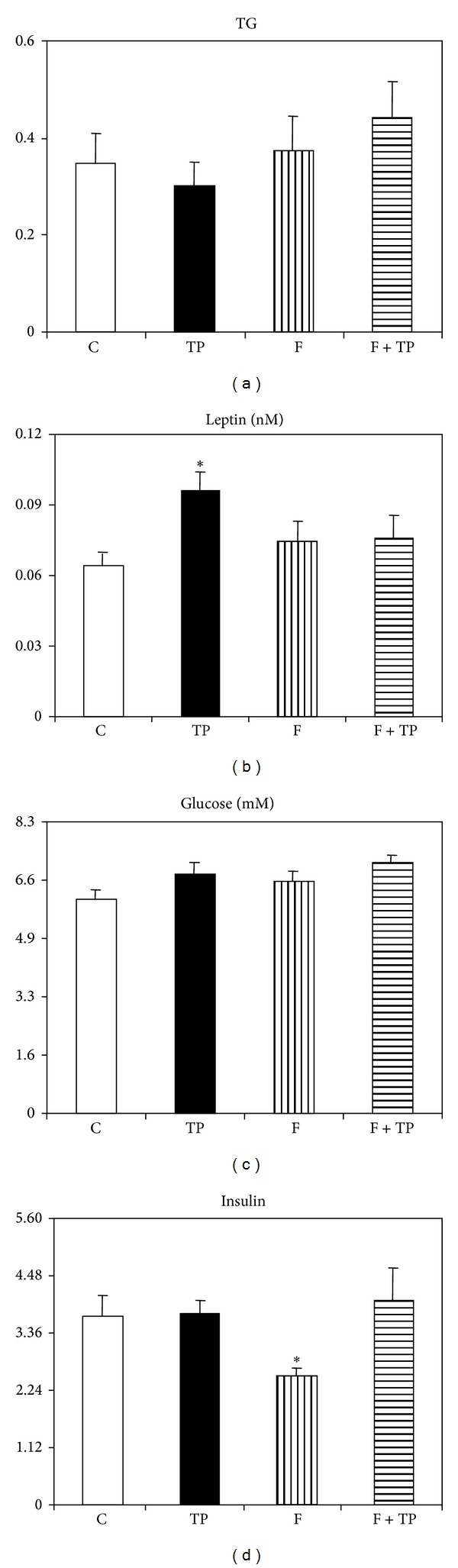
Circulating levels of triglycerides (a), leptin (b), glucose (c), and insulin (d) in different groups of juvenile rats. Values are means ± SEM (*n* = 6/7 rats per group). *P < 0.05 versus CT values.

While triglyceridemia was not modified by either neonatal treatment (Figure 2(a)); conversely, circulating leptin levels were enhanced in TP (*n* = 7) rats (*P* < 0.05 versus CT values, *n* = 6), an increase fully abolished by F cotreatment (TP + F group, *n* = 7) (Figure 2(b)). Plasma glucose levels were similar in all groups studied (Figure 2(c)). Whereas insulinemia remained at CT level in TP animals (Figure 2(d)), plasma insulin concentration was significantly (*P* < 0.05 versus CT values) reduced in F-treated animals (*n* = 6), an effect fully abolished if F rats were cotreated with androgen (TP + F group) (Figure 2(d)). Tallying with the latter data, HOMA values were significantly (*P* < 0.05) reduced in F-treated rats (0.131 ± 0.008) compared to those from CT rats (0.183 ± 0.018). The flutamide-enhanced insulin sensitivity (low HOMA score) was severely impaired by TP cotreatment (TP + F rats: 0.236 ± 0.033; *P* < 0.05 versus F values). HOMA score remained normal in TP rats (0.189 ± 0.017).

### 3.3. Steroidogenic Function

**Figure 3:**
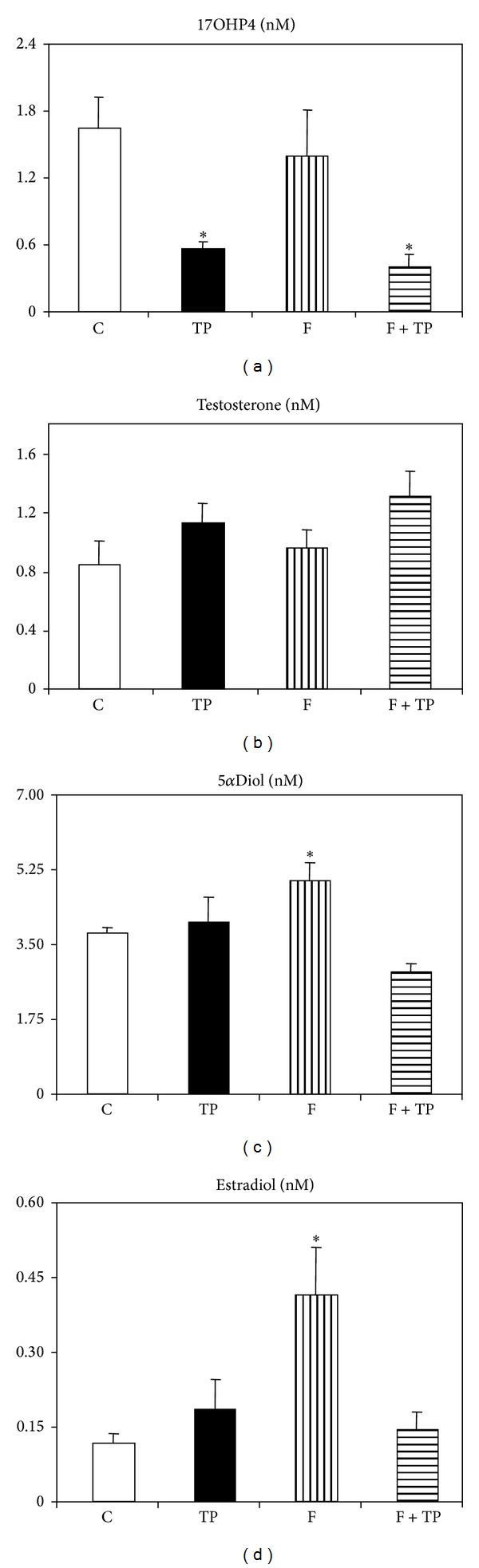
Circulating levels of 17 hydroxy-progesterone (a), testosterone (b), 5*α*-androstane-3*α*, 17*β*-diol (c), and 17*β*-estradiol (d) in different groups of juvenile rats. Values are means ± SEM (*n* = 6/7 rats per group). *P < 0.05 versus CT values.

With respect to corticoadrenal steroid contribution to the periphery, plasma, and AG corticosterone concentrations were similar in all groups (data not shown), and results were concordant with those obtained in isolated fasciculata-reticularis enriched cells incubated *in vitro* in absence or presence of ACTH (data not shown). Interestingly, the steroidogenic pathway seems to be altered at the ovarian level in animals having received androgen treatment (TP and TP + F groups; *n* = 7 rat per group); indeed, they displayed reduced (*P* < 0.05 versus CT rats, *n* = 6 rats) 17HOP4 plasma concentrations (Figure 3(a)), whereas in F-treated animals (*n* = 6) the peripheral level of this steroid remained normal (Figure 3(a)). Regarding androgens, plasma testosterone and 17*α*Diol concentrations were not modified by neonatal TP treatment (Figures 3(b) and 3(c), resp.). Conversely, while neonatal F treatment did not modify circulating testosterone levels (Figure 3(b)), it significantly (*P* < 0.05 versus CT values) enhanced 5*α*Diol plasma concentrations (Figure 3(c)); however, this effect was fully counteracted by androgen cotreatment (TP + F animals) (Figure 3(c)). Peripheral E2 concentrations were significantly enhanced in F rats (*P* < 0.05 versus CT values), an effect fully abolished by TP cotreatment (Figure 3(d)). 

**Figure 4:**
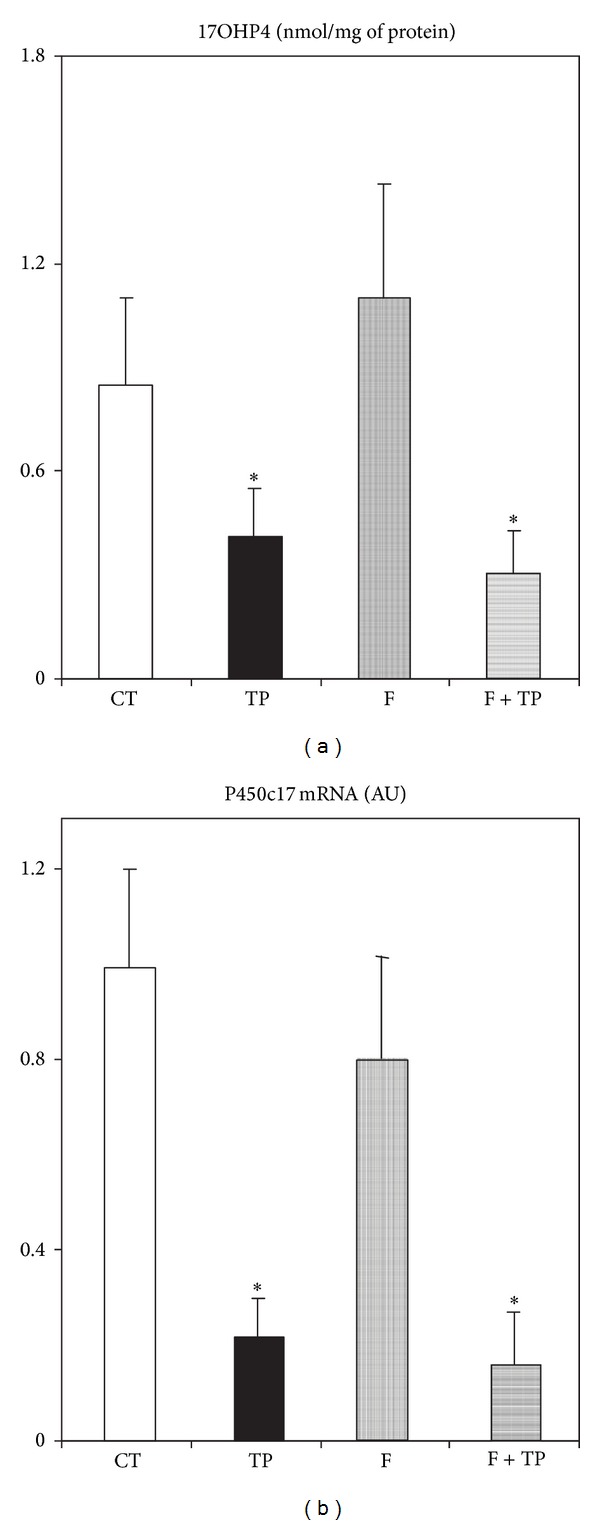
Ovarian concentration of 17 hydroxy-progesterone (a) and cytochrome P450, family 17, subfamily A, polypeptide 1 (b) mRNA in different groups of experimental rats. Values are means ± SEM (*n* = 4/5 ovaries per group). *P < 0.05 versus CT values.

The results of peripheral 17HOP4 levels agreed with data from measurements of 17HOP4 content in ovaries. Indeed, adrenal gland 17HOP4 content was detected at a very low level in all groups, with no differences among them (data not shown); conversely, in ovaries very significant differences were found (Figure 4(a)). TP-treated rats (*n* = 5) either cotreated or not with F displayed a reduced (*P* < 0.05 versus CT values, *n* = 4) 17HOP4 ovary content (Figure 4(a)). Conversely, F treatment (*n* = 4) by itself did not modify the ovarian concentration of 17HOP4 (Figure 4(a)). As expected, no P450c17 mRNA was detected in any adrenal gland examined (data not shown). Consequently, ovary P450c17 mRNA abundance was significantly (*P* < 0.05 versus CT levels) reduced in ovaries from both TP and TP + F rats, whereas it remained at CT expression level in ovarian tissues from F rats (Figure 4(b)). 

## 4. Discussion

The present study supports that neonatal modifications in androgen bioactivity clearly impacted on the organization of several metabolic and neuroendocrine functions when female rats reached juvenile age. This clearly indicates that not only the neonatal androgen excess but also a lack of full functionality of AR is involved in the physiological programming of the organism [19, 20].

 Two opposite approaches were used here to contemplate that AR is one of the structures that plays a pivotal role in the metabolic programming of the organism. Indeed, several authors including our group have demonstrated the deleterious effects of neonatal androgen excess in the physiology of the adult female rat [8, 10, 19, 21]. On the other hand, early (pre-/postnatal) flutamide intervention has been used to test the long-term effect of lack of full AR function in adult rats [13, 22, 23]. We must mention that the efficacy of flutamide, a nonsteroidal pure anti-AR [12], has largely been proved through its therapeutic effect to mitigate androgen-dependent prostate cancer [12, 24].

We presently found that, whereas flutamide-treated rats grew slowly, expectedly, androgen-treated rats grew faster [24] and that the androgen effect was corrected, at least in part, when rats were cotreated with flutamide. This androgen effect appeared to act through its anabolic activity [24]. Interestingly, animals with retarded growth (F rats) are characterized by hypophagia, thus evidencing a physiological orexigenic activity of endogenous androgen [25]. Moreover, we found that the hypophagia developed by flutamide-treated rats was no longer observed after cotreating animals with androgen (TP + F rats), thus clearly indicating an AR-mediated effect. 

New data on the neonatally androgenized rat model at juvenile age are provided here. Among these and compared to normal littermate rats, we observed (a) early disruption in the adipo insular axis function such as enhanced PMAT mass and hyperleptinemia while still managing carbohydrate (glycemia) and lipid (triglyceridemia) metabolism and (b) ovarian dysfunction. These findings indicate that, compared to the adult stage, at juvenile age the deleterious metabolic effect of early transient hyperandrogenization is not yet fully developed (e.g., enhanced triglyceridemia and insulinemia) [10]. 

We also observed that PMAT pad mass was enhanced and lowered by testosterone and flutamide treatments, respectively; remarkably, the individual effect of either compound was absent when administered to rats in a combined manner. Once again, these observations strongly argue in favor of the accepted testosterone effect on adipose tissue mass. Indeed, hyperadiposity [8–10, 19, 21] seems to be developed by transient hyperandrogenemia, even when examining regionalized fat depots. For instance, increased visceral and mesenteric fatnesses were reported in neonatally androgenized rats [8, 19] as well as in PM fat pads [10, 21]. Now demonstrated by both designs: androgen excess (TP rats) and androgen-bioactivity defect (F rats). Further, concordant with data from PMAT mass, neonatal androgenization also resulted in juvenile animals, as in older ones [10], with high leptinemia. We previously demonstrated that transient high concentrations of testosterone clearly induce a spontaneous increase in leptin secretion by adipocytes isolated from normal adult female rats [9]. Importantly, as observed in adipose tissue pad mass, enhancement in peripheral leptin levels induced by neonatal androgen treatment was fully prevented by AR-blocker cotreatment. These data suggest an androgen, AR-mediated effect on the endocrine function of adipose tissue.

As mentioned above, whereas lipidic and glucose homeostases were not affected by any neonatal treatment, other parameters examined revealed that animals neonatally blocked from AR functionality revealed improved peripheral insulin sensitivity (e.g., low insulinemia and HOMA values); however, this beneficial metabolic effect was no longer observed when animals received androgen and flutamide in cotreatment. These data fully support the concept that a sex steroid (androgen) basis is highly involved in the later development of clinical states (such as in human PCOS) of impaired insulin sensitivity dependent on early hyperandrogenism [26, 27]. Nevertheless, it must be pointed out that in favor of the metabolic advantage induced by flutamide treatment this was part of a phenotype characterized by reduced food intake and body weight. 

Finally, whereas peripheral testosterone concentrations were not affected by any neonatal treatment, increased peripheral levels of testosterone metabolites [28], 5*α*Diol, and E2 were found in F-treated rats. The latter changes were fully prevented by TP cotreatment. Although steroidogenic adrenal function was not modified by any neonatal treatment, conversely, only neonatally androgenized rats displayed a drastic reduction in 17HOP4 plasma concentrations. Concordantly, reduced ovary but not adrenal 17HOP4 concentration was found in these rats. Confirmation of these data was the finding of diminished P450c17 mRNA expression level in these ovaries. To our knowledge, this is the first report addressing impaired ovary P450c17 activity due to neonatal androgenization in the juvenile rat.

## 5. Conclusions

Neonatal manipulation of AR function in the female rat by either androgen excess or AR-blockage impacted on individual metabolic-neuroendocrine programming. These modifications seem to be perpetuated and even aggravated once rats reach reproductive age [8, 10]. Indeed, AR-blocker therapy by itself ameliorated several metabolic functions and, when used as cotreatment, prevented several dysfunctions induced by hyperandrogenemia. Thus, a safe antiandrogen therapy could be used in pregnant women displaying hyperandrogenemia/hyperandrogenism [26, 27] for further prevention of newborn's miss-programming. However, in spite of the severe ovarian dysfunction induced by neonatal hyperandrogenemia, it remains to be further investigated whether flutamide is fully able to cross the blood brain barrier [29] and whether the deleterious androgen excess impact on the reproductive axis is truly irreversible [30].
